# Screening For Yeast Phytase Leads to the Identification of a New Cell-Bound and Secreted Activity in *Cyberlindnera jadinii* CJ2

**DOI:** 10.3389/fbioe.2021.662598

**Published:** 2021-05-24

**Authors:** Claudia Capusoni, Immacolata Serra, Silvia Donzella, Concetta Compagno

**Affiliations:** Department of Food, Environmental and Nutritional Sciences, University of Milan, Milan, Italy

**Keywords:** phytic acid, yeast, *Cyberlindnera jadinii*, feed additive, food production, phytase

## Abstract

Phytic acid is an anti-nutritional compound able to chelate proteins and ions. For this reason, the food industry is looking for a convenient method which allows its degradation. Phytases are a class of enzymes that catalyze the degradation of phytic acid and are used as additives in feed-related industrial processes. Due to their industrial importance, our goal was to identify new activities that exhibit best performances in terms of tolerance to high temperature and acidic pH. As a result of an initial screening on 21 yeast species, we focused our attention on phytases found in *Cyberlindnera jadinii*, *Kluyveromyces marxianus*, and *Torulaspora delbrueckeii*. In particular, *C. jadinii* showed the highest secreted and cell-bound activity, with optimum of temperature and pH at 50°C and 4.5, respectively. These characteristics suggest that this enzyme could be successfully used for feed as well as for food-related industrial applications.

## Introduction

Phytic acid (myo-inositol 1,2,3,4,5,6-hexakis dihydrogen phosphate) is the main source of stored phosphorus in grains, oil seeds, and nuts (Mullaney and Ullah, 2003), typically representing up to 60–80% of total phosphorus in seed, and playing an important role during seed germination and growth (Shi et al., 2005). The presence of phytic acid creates problems in breeding, being feeds mainly composed by vegetal materials rich in this acid. Polygastric animals are able to degrade phytate, thanks to their particular gut microbiota (Nakashima et al., 2007), but this process does not occur in the monogastric ones, like poultry, pigs, fishes, and also humans. Since phytate cannot be metabolized, feed for monogastric animals are often fortified with inorganic phosphorus, increasing their final cost. In addition, accumulation of phytic acid has a negative effect on animal health, because it represents an anti-nutritional and chelating agent, that reduces bioavailability of proteins and ions like Fe^3+^, Ca^2+^, Zn^2+^, and Mg^2+^ forming insoluble complexes (Reddy et al., 1982; Coban and Demirci, 2017). The undigested phytate then accumulates in manure and liquid effluents, leading to phosphorus pollution and water eutrophication. Also for human nutrition, there is now an increasing attention about these aspects. Phytate degradation in food is mediated mainly by fermentation processes led by phytate-degrading microorganisms (De Angelis et al., 2003; Rizzello et al., 2010) or during the food processing by endogenous phytases present in food matrix (Leenhardt et al., 2005). For the reasons described above, science community has focused attentions on phytate-degrading enzymes (Lei et al., 2013; Mrudula Vasudevan et al., 2019; Pires et al., 2019).

Phytases are a class of enzymes that catalyze the hydrolytic degradation of phytic acid to free inorganic phosphorus, to yield lower myo-inositol phosphate esters and, in some case, free myo-inositol (Vats and Banerjee, 2004). Enzymes described as phytases show different structures: histidine acid phosphatase (HAP), β propeller phytase (BPP), and purple acid phosphatase (PAP) (Mullaney and Ullah, 2003). The most known and wide class is HAPs (EC 3.1.3.8). This class is ubiquitous, indeed HAPs can be found not only in bacteria, yeasts, and filamentous fungi but also in upper eukaryotes (Lei et al., 2013). All the proteins belonging to this class maintain two common domains: a conserved N-terminal heptapeptide active site RHGXRXP (aa 38–44) and a C-terminal catalytically active dipeptide HD (aa 325–326) (Mullaney and Ullah, 2003). Among HAPs exist a variety of specific activities. Wyss and coworkers analyzed several fungal phytases dividing them in two different subclasses: one with broad substrate specificity but low specific activity on phytic acid (PhyBp), and the second with narrow substrate specificity but high activity on phytic acid (PhyAp) (Wyss et al., 1999). Curiously, some organisms as *Aspergillus niger* possess both forms (Mullaney and Ullah, 2003).

Although phytases have been reported in a wide range of bacteria, not many of them have been used so far as feed supplement, since their neutral/alkaline pH optimum and their optimal temperature could preclude their activity in these processes. For industrial applications, the ideal phytase should display in fact three characteristics: ability to hydrolyze phytic acid in the upper digestive tract of the animals, resilience up to 65–90°C and cheap production cost. In particular, to work properly in the digestive tract, phytase needs to have a pH optimum between 3.5 and 5.5 and optimum of temperature in the range 37–40°C. Furthermore, phytases should actively work also at higher temperatures, required in feed production processes, as during pelletting and heat treatment to control *Salmonella* spoilage (Mrudula Vasudevan et al., 2019). In addition, it would need to be resistant to protease activity and to show low sensitivity to ions (Wyss et al., 1999).

Addition of phytases is currently not yet applied in human food production for increasing the mineral bioavailability of the food. This is mainly due to the fact that, so far, all the commercial phytase-producing organisms are genetically modified organisms (GMOs), which are commonly not well accepted for human food production.

Yeasts are good candidates for phytase production and some of them have been already characterized (Ragon et al., 2009; Nuobariene et al., 2011; Greppi et al., 2015; Pires et al., 2019). These enzymes show different localizations: in some species, phytases are extracellular enzymes, in others are cell bound or released in the periplasmic space (In et al., 2009; Olstorpe et al., 2009; Kaur et al., 2010; Hellström et al., 2015; Kłosowski et al., 2018).

With the aim to find new phytases with characteristics suitable for feed and food industrial applications, in this study, we screened 28 yeast strains isolated from different environments, terrestrial and marine. We investigated cellular localization of phytase activity and, for some of them, the effects of different phosphorus sources on their expression. In addition, we characterized optimal temperature and pH.

## Materials and Methods

### Yeast Strains

The yeast strains studied in this work (Table 1 and Supplementary Table 1) belong to CBS collection, UBO Culture Collection (UBO-CC^1^), DBVPG collection, and private collections at the University of Milan (CML and UMY). Yeasts were stored in YPD-20% (vol/vol) glycerol stocks at −80°C.

**TABLE 1:** Results of phytase screening performed on 28 yeast strains grown on medium containing phytic acid as sole phosphorus source.

**Yeast**	**Strain**	**OD_600 *nm*_**	**Cell-bound**	**Extracellular**
*Debaryomyces hansenii*	MI 1	5.5	BDL	BDL
*Ciberlindnera jadinii*	CJ2	28	YES	YES
*Schizosaccharomyces pombe*	Y709	4	BDL	BDL
*Kluyveromyces lactis*	CBS 2359	3	YES	BDL
*Kluyveromyces lactis*	Y1356	1	BDL	BDL
*Kluyveromyces marxianus*	Y1058	14	YES	BDL
*Hanseniaspora uvarum*	UMY 514	8	YES	BDL
*Hanseniaspora uvarum*	UMY 571	14	YES	BDL
*Saccharomyces cerevisiae*	CENPK 113 7D	7.78	BDL	BDL
*Saccharomyces cerevisiae*	LALVIN T73	8.36	BDL	BDL
*Brettanomyces bruxellensis*	CBS 2499	10	YES	BDL
*Lachancea thermotolerans*	CBS 6340	8	YES	BDL
*Torulaspora delbruekii*	CBS1466	8.56	YES	BDL
*Candida humilis*	CBS 5658	8.6	BDL	BDL
*Candida milleri*	CBS 6897	11	BDL	BDL
*Rhodosporidium azoricum*	DBVPG 4620	12	BDL	BDL
*Zygosaccharomyces kombutchaensis*	CBS 8849	7	YES	BDL
*Kazakistania unispora*	CML133	3	BDL	BDL
*Meyerozyma guilliermondii*	UBOCC-A-214008	26	YES	BDL
*Meyerozyma guilliermondii*	UBOCC-A-214143	20	YES	BDL
*Pichia guilliermondii*	EX15 UBOCC-A-208004	21	YES	BDL
*Rhodotolura mucilaginosa*	UBOCC-A-214025	10	BDL	BDL
*Rhodotolura mucilaginosa*	UBOCC-A-214036	7.9	YES	BDL
*Candida atlantica*	Mo31 UBOCC-A-208026	25	YES	BDL
*Candida oceani*	Mo39 UBOCC-A-208034	4.2	BDL	BDL
*Debaryomyces hansenii*	BIO2 UBOCC-A-208002	11	YES	BDL
*Debaryomyces hansenii*	Mo40 UBOCC-A-208035	8.2	BDL	BDL
*Rhodotorula diobovata*	Mo38 UBOCC-A-208033	12.5	YES	BDL

*References for strains origin are reported in Supplementary Table 1. BDL, below detection limit.*

### Media and Cultivation

Yeasts were cultivated on different media.

YPD: 10 g/L yeast extract, 20 g/L peptone, 20 g/L glucose.

MMPhy: 20 g/L glucose, 5 g/L (NH_4_)_2_SO_4_, 0.5 g/L MgSO_4_^∗^7H_2_0, 0.11 g/L phytic acid sodium salt hydrate (Sigma Aldrich, Milano, Italy), trace metals (di-sodic EDTA 15 mg/L, ZnSO_4_^∗^4H_2_O 4.5 mg/L, MnCl^∗^4H_2_O 0.1 mg/L, CoCl_2_^∗^6 H_2_O 0.3 mg/L, CuSO_4_^∗^5H_2_O 0.3 mg/L, Na_2_MoO_4_^∗^2H_2_0 0.4 mg/L, CaCl_2_^∗^2H_2_O 4.5 mg/L, FeSO_4_^∗^7H_2_O 3 mg/L, H_3_BO_3_ 1 mg/L, KI 0.1 g/L) and vitamins, d-biotin 0.05 mg/L, calcium D-pantothenate 1 mg/L, nicotinic acid 1 mg/L, myoinositol 25 mg/L, thiamine hydrochloride 1 mg/L, pyridoxine hydrochloride, *p*-aminobenzoic acid 0.2 mg/L) as reported in Merico et al. (2007) with some modification.

MM-: 20 g/L glucose, 5 g/L (NH_4_)_2_SO_4_, 0.5 g/L MgSO_4_^∗^7H_2_O, trace metals, and vitamins as in MMPhy.

MMP: 20 g/L glucose, 5 g/L (NH_4_)_2_SO_4_, 0.5 g/L MgSO_4_^∗^7H_2_O, KH_2_PO_4_ 1 g/L, trace metals, and vitamins as in MMPhy.

MMP/Phy: 20 g/L glucose, 5 g/L (NH_4_)_2_SO_4_, 0.5 g/L MgSO4^∗^7H_2_O, 0.11 g/L phytic acid sodium salt hydrate, KH_2_PO_4_ 1 g/L, trace metals, and vitamins as in MMPhy.

In all media, pH was adjusted to 4.5 using H_2_SO_4_.

Yeast cells were cultivated at 28°C in a rotary shaker at 150 rpm in bluffed flasks (100 ml) containing 20 ml of medium. Optical density was monitored at 600 nm (OD_600_). Cells were precultured for 24 h in YPD, harvested by centrifugation at 5,000 rpm, and washed three times with sterile NaCl solution (9 g/L). Then they were used to inoculate alternatively MMPhy, MMP, MMP/Phy, and YPD at an initial OD_600_ = 1 (corresponding to 10^7^ cells/ml approximately).

### Dry Weight Determination

For dry weight measurements (DW), samples from different culture conditions were collected (in triplicate at each point). Cells were filtered through a glass microfiber GF/A filter (Whatman), washed with three volumes of deionized water and dried at 100°C for 24 h.

### Phytase Activity Determination

Enzymatic activity was determined on supernatants (for extracellular activity) and whole cells (for cell-bound activity) of early exponential phase cultures. The activity was measured by ortho-phosphate production, following ammonium molibdate blue method as reported by Schimizu (Shimizu, 1992), with some modifications.

For extracellular activity determination, cell cultures were centrifuged at 13,000 rpm and 1 ml of supernatant was added to 4 ml of buffer composed by 0.2 M Na acetate/acetic acid, 8 mM phytic acid at pH 4.5. To determine cell-bound activity, a standard amount of cells (corresponding to 10 mg of dry weight) was collected, washed twice with 0.2 M Na acetate/acetic acid at pH 4.5, and resuspended in a final volume of 1 ml of water. Cell suspension was added to 4 ml of buffer in 0.2 M Na acetate/acetic acid, 8 mM phytic acid at pH 4.5. All buffers employed to test enzymatic activity were prewarmed at the reaction temperature. Blank was assembled using 1 ml of water and 4 ml of 0.2 M Na acetate/acetic acid at pH 4.5, 8 mM phytic acid, and treated as sample.

For enzymatic activity determination, 5 ml of reaction mixture were incubated in 15 ml tube at 37°C and stirred at 300 rpm. The reaction was immediately stopped (for time 0) and then stopped after 15, 30, 60, and 120 min by mixing 0.5 ml of reaction mixture with 0.5 ml of TCA 5% solution. Samples were centrifuged for 3 min at 13,000 rpm and the supernatants collected. To determine orthophosphate concentration, 0.4 ml of supernatant was added to 0.4 ml of molibdate solution. This solution was prepared daily, by mixing solution A and B in a ratio of 4:1 (solution A: 2.6% N_6_H_24_Mo_7_O_24_^∗^4H_2_O and 5.5% H_2_SO_4_; solution B: 4.6% FeSO_4_^∗^7H_2_O). The sample was incubated 10 min at 25°C and read against blank at OD_700_. Phosphate concentration was determined using a standard curve for KH_2_PO_4_. One unit of phytase is defined as the amount of protein that hydrolyses 1 μmol of phosphorus/min. Specific activity is expressed as milliunits per milligram of cell dry weight. To determine the effect of temperature, samples prepared with prewarmed buffer (pH 4.5) were incubated at 50 and 60°C. To determine the effect of pH on enzymatic activity, pH buffers were adjusted at pH 4 and pH 5.5, and the reactions incubated at 37°C.

### Genomic Extraction

To isolate genomic DNA, pellets corresponding to 30 OD of cells were resuspended in 0.5 ml of 50 mM Tris–HCl, 20 mM M EDTA at pH 7.5. This suspension was transferred to a precooled tube with an equal volume of glass beads (425–600 μm). Mechanical lysis was performed using a TissueLyser LT (Qiagen) alternating 2 min of agitation at 50 Hz with 1 min in ice for four cycles. The supernatant was added with 25 μl of SDS 20% (*w*/*v*) and incubated at 65°C for 30 min. Immediately, 0.2 ml of 5 M potassium acetate was added and the tubes were placed on ice for 30 min. Samples were centrifuged at 13,000 rpm for 5 min and supernatants transferred to a fresh microcentrifuge tube. The DNA was precipitated by adding 1 vol of isopropanol. After incubation at room temperature for 5 min, the tubes were centrifuged for 10 min. The DNA was washed with 70% ethanol and dissolved in 50 μl of TE RNAse (10 mM Tris–HCl, 1 mM EDTA, pH 7.5 RNAse 100 μg/ml). Samples were incubated at 37°C for 30 min.

### Strain Identification

gDNA was amplified with Phusion taq polymerase employing universal primers for amplification on D1/D2 domain of the 26S rDNA (NL1: GCATATCAATAAGCGGAGGAAAAG, NL4: GGTCCGTGTTTCAAGACGG) 0.2 μM each, 200 μM dNTP, and MgCl_2_ 2.5 mM (Fliegerová et al., 2006). PCR amplification was carried out by denaturing at 98°C for 7 min, followed by 30 cycles of denaturing at 98°C for 30 s, annealing at 52°C for 30 s, extension at 72°C for 1 min, and a final extension at 72°C for 5 min. The produced PCR amplicon was sequenced using the Sanger method at Microsynth Seqlab (Germany), and the strain was identified by the sequence similarity using basic local alignment search tool against the NCBI databases^2^.

### Phylogenetic and Bioinformatics Analysis

Phytase sequence of *D. hansenii* Mo40 and Bio2 were obtained in this work. Phytase gene was amplified from gDNA using primers: Forward: Phy1 CCGACCATGGATGGTATCGATTTCC, Reverse: Phy2 CATCGGATCCTAATT GTCACCGGA. Primers were designed based on *D. hansenii* CBS 767 (GeneID: 2900382; XP_460696.1). PCR amplification was carried out by denaturing at 98°C for 7 min, followed by 30 cycles of denaturing at 98°C for 10 s, annealing at 59°C for 30 s, extension at 72°C for 45 s, and a final extension at 72°C for 10 min. PCR amplicons were sequenced using the Sanger method at Microsynth Seqlab (Germany).

The aminoacidic sequences (accession numbers on Supplementary Table 2) were identified through a BLASTp by XP_460696.1 of *D. hansenii* CBS 767 against the NCBI databases. For phylogenetic analysis, multiple alignments of aminoacidic sequences were performed using MUSCLE (EMBL-EBI tool on^3^) and a maximum likelihood tree was built using Mega X 10.1.7^4^. Analysis of signal secretion sequence was performed employing SignalIP-5.0 available on http://www.cbs.dtu.dk/services/SignalP/.

## Results and Discussion

### Growth in Presence of Phytic Acid as Sole Phosphorus Source

Twenty-one yeast species (28 strains) belonging to *Debaryomyces*, *Cyberlindnera*, *Schizosaccharomyces*, *Kluyveromyces*, *Saccharomyces*, *Brettanomyces*, *Candida*, *Torulaspora*, *Rhodosporidium*, *Meyerozyma*, *Hanseniaspora*, *Pichia*, *Lachancea*, *Kazakistania*, and *Rhodotorula* genera were characterized for their ability to grow using phytic acid as sole phosphorus source (Table 1 and Supplementary Figure 1). Strain CJ2 was identified in this work as *Cyberlindnera jadinii*. All strains were cultivated on medium MMPhy, and their growth was monitored for 72 h. In parallel, as negative control, growth on MM- (without any phosphorus source) was carried out, and no appreciable growth was detected (data not shown). We avoided to perform screening on solid medium due to ambiguous results reported sometimes in literature.

All strains except *Kluyveromyces lactis* Y1356 were able to grow using phytic acid as sole phosphorus source, but with variable extent (Table 1). Some strains, like *C. jadinii* and *Meyerozyma guilliermondii* were able to exceed 20 OD after 24 h of incubation, reaching 26 OD and 25 OD, respectively, after 48 h. Other strains reached lower OD values after 72 h of incubation, and *K. lactis* CBS 2359 duplicated only two times reaching 4 OD (Table 1 and Supplementary Figure 1). These differences reflect species-dependent efficiency of phytase activity, as well as species-specific mechanism of phytic acid hydrolysis. Literature reports that in some yeasts, like *Debaryomyces castellii*, phytase is able to completely hydrolyze phosphate from phytic acid (Ragon et al., 2008), but others, like in *Kodamaea ohmeri* are, on the contrary, not able to perform a complete hydrolysis of this acid, leaving some phosphate group not bioavailable (Li et al., 2009).

### Screening for Phytase Activity

In order to identify enzymes that show characteristics suitable for feed/food industrial processes, meaning a phytase able to work in the upper digestive tract of monogastric animals, with optimal temperature range between 37 and 40°C and pH range between 3.5 and 5.5, we decided to screen phytase activity at 37°C and pH 4.5. Extracellular and cell-bound activity was assayed on cells grown using phytate as sole phosphorus source, on MMPhy medium. To correctly compare phytase activity in various strains, we expressed the activity as milliunits per milligram d.w., instead of milliunits per milliliter as often reported in literature. In this way, we avoided the bias due to different ability of strains to grow in presence of phytate (Table 1), reaching different amount of biomass per milliliter.

**FIGURE 1:**
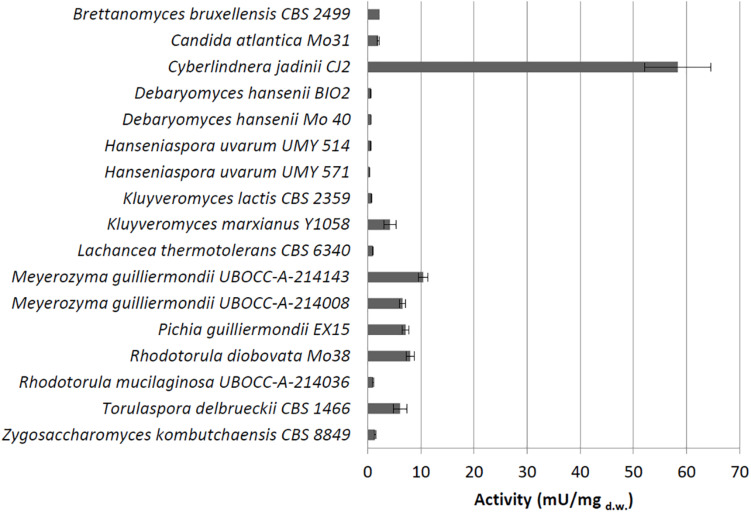
Cell-bound phytase activity (mU/mg_d.w._) detected using whole cells grown on medium containing phytate.

Under these conditions, we detected extracellular phytase activity only in *C. jadinii* (26.25 mU/mg_d.w._). On the other hand, 16 out of the 28 tested strains showed a detectable cell-bound activity (Figure 1), and *C. jadinii* was identified as the one with the highest (58.36 mU/mg_d.w._). Lower levels of cell-bound activity were found in fact in the other species (Figure 1), like *Kluyveromyces marxianus* (4.17 mU/mg_d.w._), *Meyerozyma guilliermondii* (10.49 mU/mg_d.w._), *Pichia guillermondii* (7.1 mU/mg_d.w._), *Rhodotorula diobovata* (7.99 mU/mg_d.w._), and *Torulaspora delbrueckii* (6.1 mU/mg_d.w._).

### Analysis of Conserved Domain

Aminoacidic sequences of phytase were recovered from RefSeq/GenBank database on https://www.ncbi.nlm.nih.gov/, except for *D. hansenii* Mo40 and Bio2, which were sequenced in this work. All strains analyzed possess a protein that shows a good homology with phytase of *D. hansenii* Mo40 and Bio2, and some of them, like *M. guilliermondii*, *C. jadinii*, and *K. marxianus*, exhibit even two sequences encoding for this enzyme. In the case of *K. unispora*, we could not identify any sequence even if this strain can make a duplication on MMPhy medium. Probably, this ability is due to the presence of a phosphatase that does not specifically cleave phosphate from phytic acid (Table 1).

**FIGURE 2:**
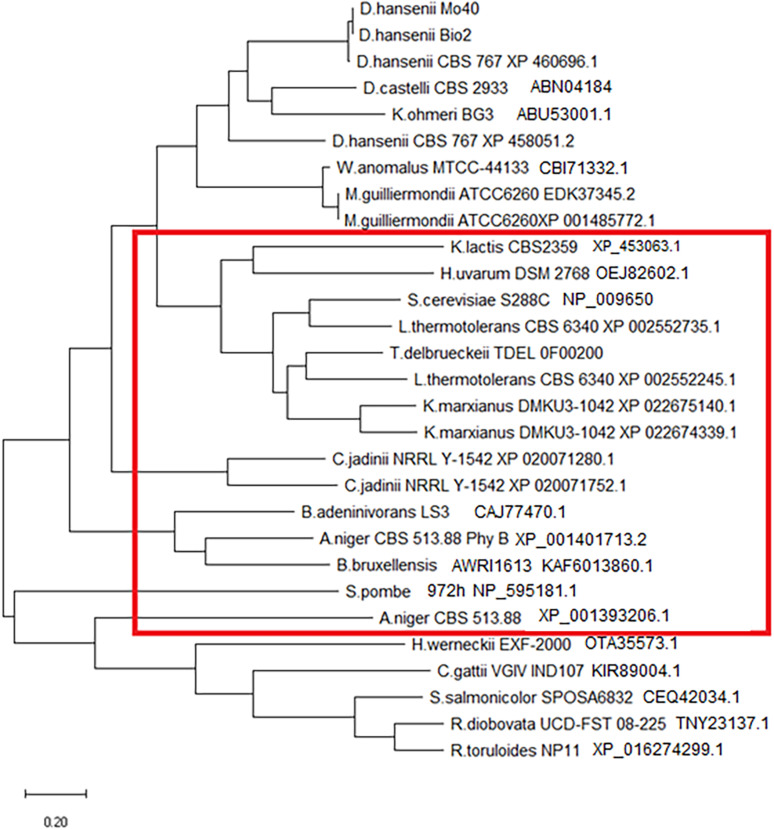
Phylogenetic tree of phytase aminoacidic sequences. The red rectangle includes proteins that contain a signal sequence for secretion (detected using SignalIP-5.0).

Generally, HAP phytase brings two common domains: a conserved N-terminal heptapeptide active site RHGXRXP (aa 38–44) and a C-terminal catalytically active dipeptide HD (aa 325–326) (Mullaney and Ullah, 2003). In the analyzed phytase sequences, the C-terminal domain is always conserved. N-terminal domain is maintained in all except in *R. toruloides*, in which the active site shows mismatch XHGHRXP, leading us to conclude that all belong to HAP family. These sequences were used to build a phylogenetic tree (Figure 2). In the red box, we included sequences that contain a signal peptide for protein secretion. Literature data (Li et al., 2009) report that *K. ohmeri* enzyme contains this sequence, but the tool we used was not able to recognize it. As reported in Figure 2, all sequences showing a signal peptide can be clustered, suggesting that extracellular localization can be phylogenetically related. The fact that we detected extracellular activity only in *C. jadinii*, could probably depend on the use of culture supernatants without any step to concentrate it. It is possible to speculate that extracellular phytase activity in other strains was too low to be detected. This hypothesis is corroborated also by studies that report in other species extracellular activity detected only in concentrated samples (Olstorpe et al., 2009; Ragon et al., 2009; Hellström et al., 2015).

### Regulation of Phytase Expression

To carry out this investigation, we selected three species, *C. jadinii*, *K. marxianus*, and *T. delbrueckii* that showed high phytase activity and could be interesting for food-related applications. *C. jadinii* and *K. marxianus* are in fact included in QPS EFSA list (Koutsoumanis et al., 2019), and *T. delbrueckii* is a wine starter with commercial name (BIODIVA^TM^—Lallemand).

Phytase activity was analyzed by cultivating yeast cells in the presence of different phosphorus sources: phytic acid (in MMPhy medium), phosphate salt (KH_2_PO_4_ in MMP medium), mixture of phytic acid and phosphate salt (in MMP/Phy medium), and also in rich medium (YPD). This allowed to understand the regulation of phytase expression based on the type of phosphate available (Table 2).

**TABLE 2:** Effects of phosphorus source on phytase activity.

	**YPD**	**MMPhy**	**MMP**	**MMP/Phy**
*C. jadinii*	2.02 ± 0.4	58.36 ± 6.24	BDL	BDL
*T. delbrueckii*	2.39 ± 0.29	6.1 ± 1.25	BDL	BDL
*K. marxianus*	1.71 ± 0.35	4.17 ± 1.17	BDL	BDL

*Cell-bound activity (mU/mg_d.w._) was assayed at 37°C, pH 4.5. BDL, below detection limit.*

When *C. jadinii* cells were cultivated in medium containing phytic acid as the sole phosphorus source, we detected 58.36 mU/mg_d.w._ of cell-bound activity (Table 2) and 26.25 mU/mg_d.w._ of extracellular activity. These activities were not appreciable when inorganic phosphate was the only source (MMP), suggesting that under this condition, the enzyme was not expressed. In addition, the concomitant presence of phytic acid and KH_2_PO_4_ (MMP/Phy medium) was not able to induce phytase activity (Table 2). On the contrary, when *C. jadinii* was cultivated in the presence of organic phosphate (YPD medium), a reduced level of cell-bound activity was detected: it decreased in fact from 58.36 mU/mg_d.w._, measured in MMPhy, to 2.02 mU/mg_d.w._ in YPD (Table 2). Under this condition, extracellular activity was under the detection limit.

The same behavior was observed in T. *delbrueckii* and *K. marxianus*. The highest activities (6.1 and 4.17 mU/mg_d.w._, respectively) were detected in the presence of sole phytic acid and decreased in YPD (Table 2). As observed in *C. jadinii*, also in *T. delbrueckii* and in *K. marxianus*, the presence of phosphate inhibited expression of phytase, being no activity detectable in cells growing in MMP as well as in MMP/Phy.

In conclusion, a high level of phytase activity can be expressed only when cells grow using phytate as sole phosphorus source. On the contrary, the presence of inorganic phosphate completely inhibits expression of phytase. This happens in fact also in medium with concomitant presence of phosphate and phytic acid. This indicates that sole presence of phytic acid is not enough to induce phytase activity, and lead us to conclude that lack of inorganic phosphate is requested for its expression. The presence of low activity in cells grown in medium not containing phosphate salt, namely YPD, corroborates this hypothesis.

An analogous phenomenon has been observed previously by Olstorpe and colleagues in 2009 (Olstorpe et al., 2009). They reported that in some *Candida* species, in *Pichia anomala* and in *S. cerevisiae*, phytase activity was repressed in presence of inorganic phosphate, but this did not occur in other species like *Arxula adeninivorans*, as well as in *Cryptococcus laurentii* (Pavlova et al., 2008). By employing mutagenesis, an improved strain with reduced phosphate repression was obtained in *Pichia kudriavzevii* (Qvirist et al., 2017). Understanding which role plays phosphorus source on expression of phytase activity is pivotal for set-up of industrial processes, due to the fact that feed/food matrices as well as cultivation media based on agrifood wastes can contain different types of this nutrient.

### Characterization of Phytase Activity

Phytases suitable for feed/food-related processes need to work under conditions present in the upper digestive tract of monogastric animals, and/or need to be resilient during production processes. To work in the digestive tract, a good phytase should exhibit a pH optimum between 3.5 and 5.5 and high activity at 37°C. Resilience at higher temperature can be requested because heat treatments are commonly adopted to contain spoilage and during pelleting processes. This treatment permits also the incorporation of ingredients in the feed to “lock” the feed mixture. Unfortunately, heat treatment could reduce phytase activity, and for this reason, it is important to select a thermostable enzyme (Mrudula Vasudevan et al., 2019).

**FIGURE 3:**
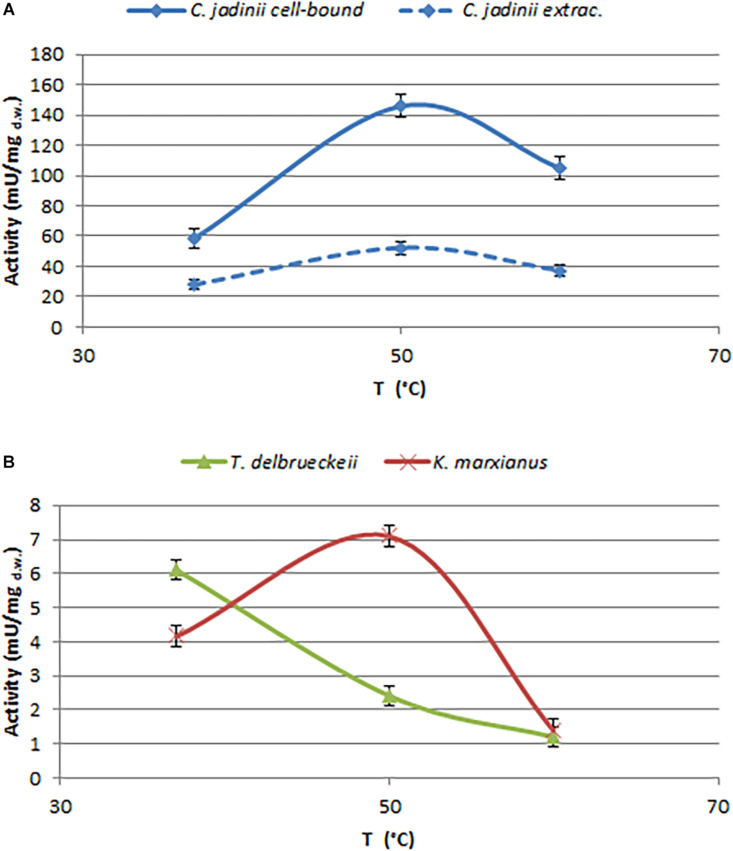
Effects of temperature on phytase activity, measured at pH 4.5. **(A)** Cell-bound (full line) and extracellular (dotted line) activity detected on *C. jadinii*. **(B)** Cell-bound activity detected on *T. delbruekii* and *K. marxianus*.

**FIGURE 4:**
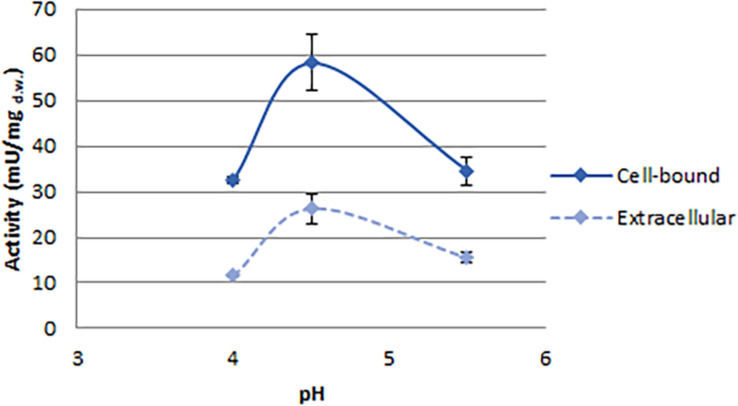
Effect of pH on phytase activity detected in *C. jadinii*. Activity was measured at 37°C.

In order to select enzymes suitable for these applications, the effects of temperature and pH were investigated (Figures 3, 4). The optimal temperature for phytase activity in *C. jadinii* was found to be 50°C. When the assay was performed at this temperature, cell-bound activity reached 146 mU/mg_d.w._ and extracellular activity was 51.95 mU/mg_d.w._ At 60°C, the values for cell-bound and extracellular activity were lower: 105.2 and 37.2 mU/mg_d.w._, respectively (Figure 3A). Similar results were obtained for *K. marxianus*. Also in this case, the optimum of temperature was observed at 50°C, with activity of 7.11 mU/mg_d.w._ A similar temperature activity profile was found for phytase from *Rhodotorula mucilaginosa* JMUY14 (Yu et al., 2015). On the other hand, in *T. delbrueckii*, the highest activity of 6.1 mU/mg_d.w._ was detected at 37°C (Figure 3B). Data reported in Figure 3 lead us to hypothesize that phytase activity in *C. jadinii* could be suitable for industrial purpose. High phytase activity detected even at 60°C suggests that this enzyme could be resilient also at high temperatures used during feeds production. In addition, at 37°C *C. jadinii* phytase activity is higher in comparison with *K. marxianus* and *T. delbrueckii* ones.

With the aim to investigate the effect of pH on *C. jadinii* activity, we selected temperature of 37°C (Figure 4). Even if this temperature is not the optimum for *C. jadinii*, it is the one requested to work at gastric level. As reported in Figure 4, pH optimum for this phytase was 4.5, similarly to phytases found in other yeasts (In et al., 2009; Caputo et al., 2015; Ogunremi et al., 2020). As reported by Lei et al. (2013), this is an important characteristic for the development of enzymes as feed/food additive.

Comparing our results with some data reported in literature, it is possible to observe that phytase activity found in *C. jadinii* could be promising for future applications (Supplementary Table 3). To the best of our knowledge, *C. jadinii* cell-bound activity is one of the highest observed on cells grown in mineral media with phytate as sole phosphorus sources.

In conclusion, we think that these results found for *C. jadinii* phytase activity could represent a good starting point to set-up optimization of cultural conditions, in order to improve phytase production. As reported, with a statistical approach for medium optimization, phytase production can be easily increased (Puppala et al., 2018). The productivity of phytase using *Aspergillus niger* under submerged fermentation conditions was improved by 3.97 times employing a statistical media optimization strategy and Box-Behnken experimental designs (Shah et al., 2017). Optimization of temperature, pH, and aeration allowed the successful production of phytase with *A. ficuum* in submerged fermentation as opposed to the traditional solid-state fermentation (Coban and Demirci, 2014). In *S. cerevisiae* modulation of media components, like addition of magnesium sulfate, manganese sulfate, and ferrous sulfate, and scaling up in 10 L fermenter could increase phytase activity from 45 to 164 mU/mg_d.w._. In *K. marxianus*, phytase activity could be easily increased adding to fermentation media cheap substrates rich in phytic acid like rice bran (Pires et al., 2019). Similar behavior was observed in *W. anomalus*, where the presence of cane molasses in media can increase enzymatic activity from 6 up to 176 mU/mg_d.w._ reducing enzyme production cost from 0.25 to 0.006 £/1,000 U (Vohra and Satyanarayana, 2004). In our case, performing media optimization could be the right approach in order to increase phytase productivity, reducing its production cost.

Furthermore, Cruz and colleagues demonstrated that biomass of *C. jadinii* can partially replace feed protein content (generally consistent in soybean meal, fish meal, rapeseed meal) in swine and poultry formulation (Cruz et al., 2020a,b). The possibility to have active enzymes in feed could be very appealing in order to decrease their phytate content. This phenomenon has been observed in *W. anomalus*, whose biomass-containing phytase was added to aquaculture feed (1,000 U/kg feed), with results comparable with commercial phytase (Vohra et al., 2011). The safety statement of *C. jadinii* could open the possibility for applications of this no-GMO microorganism also in food-related processes to produce functional foods (Handa et al., 2020).

## Conclusion

The combined effect of phytate as antinutritional factor and as cause of environmental pollution makes phytase an industrially interesting target. We identified new phytase activities in “safe” yeasts, like *C. jadinii*, *K. marxianus*, and *T. delbrueckii*. In particular, *C. jadinii* shows the highest enzymatic activity localized both extracellularly and cell-bound. Our results suggest that this phytase is suitable as additive in feed/food-related processes. Indeed, its activity showed characteristics in terms of temperature and pH suitable to work efficiently under conditions compatible with the upper digestive tract of monogastric animals as well as to be used in feed industrial production processes. The safety statement of *C. jadinii* could open the possibility for its application to reduce phytate content in food matrices.

Furthermore, a released phytase significantly increases the interaction between phytate present in food matrix and the enzyme. In addition, an extracellular enzyme improves greatly the downstream during industrial production.

## Data Availability Statement

The original contributions presented in the study are included in the article/Supplementary Material, further inquiries can be directed to the corresponding author/s.

## Author Contributions

ClC: investigation and writing the original draft. IS: investigation and review and editing the draft. SD: investigation. CoC: conceptualization, supervision, and review and editing the draft. All authors contributed to the article and approved the submitted version.

## Conflict of Interest

The authors declare that the research was conducted in the absence of any commercial or financial relationships that could be construed as a potential conflict of interest.

## Footnotes

1 http://www.univ-brest.fr/ubocc

2 http://www.ncbi.nlm.nih.gov/BLAST/

3 https://www.ebi.ac.uk/Tools/msa/muscle/

4 www.megasoftware.net

## Abbreviations

aa: aminoacid
EDTA: ethylene-diamine-tetraacetic acid
OD: optical density
TCA: tricloracetic acid
gDNA: genomic DNA
U: unit.
