# Supplementary material for: Screening For Yeast Phytase Leads to the Identification of a New Cell-Bound and Secreted Activity in Cyberlindnera jadinii CJ2
Source: Front Bioeng Biotechnol. 2021 May 24;9:662598. doi: 10.3389/fbioe.2021.662598 (PMC8181137; doi:10.3389/fbioe.2021.662598)
Supplement: Supplementary file 1 [file Data_Sheet_1.pdf]

Supplementary Material

1 Supplementary Tables

Table S1. Yeast sources and related references.

Yeasts	Strains	Source
Debaryomyces hansenii	MI 1	Creamery
Ciberlindnera jadinii	CJ2	Sugar cane bagasse (This work)
Schizosaccharomyces pombe	Y709	NA
Kluyveromyces lactis	CBS 2359	Creamery (Wésolowski et al., 1982)
Kluyveromyces lactis	Y1356	NA
Kluyveromyces marxianus	Y1058	(Hagman et al., 2015)
Hanseniaspora uvarum	UMY 514	Grapes
Hanseniaspora uvarum	UMY 571	Grapes
Saccharomyces cerevisiae	CENPK 113 7D	Lab strain (Otero et al., 2010)
Saccharomyces cerevisiae	LALVIN T73	Lallemandstarter
Brettanomyces bruxellensis	CBS2499	Wine (Piškur et al., 2012)
Lachancea thermotolerans	CBS 6340	Mirabelle-plum conserve (Zhou et al., 2019)
Torulospora delbrukeii	CBS1466	NA (Kurtzman, 2003)
Candida humilis	CBS 5658	Bantu beer (Kurtzman, 2011)
Candida milleri	CBS 6897	Sourdough for bread (Yarrow, 1978)
Rhodosporidium azoricum	DBVPG 4620	Soil (Capusoni et al., 2017)
Zygosaccharomyces	CBS 8849	Dried tea fungus (from Kombucha tea)
Kazakistania unispora	CML133	Fermented maize bran (Decimo et al., 2017)
Meyerozima guilliermondii	UBOCC-A-214008	Sediment (Rédou et al., 2015)
Meyerozima guilliermondii	UBOCC-A-214143	Sediment (Rédou et al., 2015)
Pichia guilliermondii	UBOCC-A-208004	Siliceous sponge (Burgaud et al., 2015)
Rhodotolura mucilaginosa	UBOCC-A-214025	Sediment (Rédou et al., 2015)
Rhodotolura mucilaginosa	UBOCC-A-214036	Sediment (Rédou et al., 2015)
Candida atlantica	Mo31 UBOCC-A-208026	Bathymodiolus azoricus (Burgaud et al.,
Candida oceani	Mo39 UBOCC-A-208034	Sponge (Burgaud et al., 2015)
Debaryomyces hansenii	BIO2 UBOCC-A-208002	Gastropod gills (Burgaud et al., 2010)
Debaryomyces hansenii	Mo40 UBOCC-A-208035	Coral (Burgaud et al., 2015)
Rhodotorula diobovata	Mo38 UBOCC-A-208033	Sponge (Burgaud et al., 2010)

NA: not available

S2. Aminoacidic sequences used to perform phylogenetic analysis.

Strain	Sequence ID
Debaryomyces hansenii CBS 767	XP_460696.1<br>XP_458051.2
Debaryomyces castelli CBS 2933	ABN04184
Kodamaea ohmeri BG3	ABU53001.1
Wickerhamomyces anomalus MTCC-44133	CBI71332.1
Meyerozyma guilliermondii ATCC6260	EDK37345.2<br>XP_001485772.1
Kluyveromyces lactis CBS2359	XP_453063.1
Hanseniaspora uvarum DSM2768	OEJ82602.1
Saccharomyces cerevisiae S288C	NP_009650
Lachanca thermotolerans CBS6340	XP_002552735.1<br>XP_002552245.1
Torulaspora delbrueckii CBS 1146	TDEL_0F00200
Kluyveromyces marxianus DMKU3-1042	XP_022675140.1<br>XP_022674339.1
Cyberlindnera jadinii NRRL Y-1542	XP_020071280.1<br>XP_020071752.1
Blastobotrys adeninivorans LS3	CAJ77470.1
Aspergillus niger CBS 513.88	XP_001401713.2<br>XP_001393206.1
Brettanomyces bruxellensis AWRI1613	KAF6013860
Schizosaccharomyces pombe 972h	NP_595181.1
Hortaea werneckii EXF_2000	OTA35573.1
Cryptococcus gattii VGIV IND107	KIR89004.1
Sporobolomyces salmonicolor SPOSA6832	CEQ42034.1
Rhodotorula diobovata UCD-FST 08-225	TNY23137.1
Rhodosporidium toruloides NP11	XP_016274299.1

Table S3: Phytase activity in yeasts.

Strain	Media	pH	T (°C)	Cell bound ($mU/mg_{d.w.}$)	Reference
C. jadinii CJ2	minimal media phytate	4,5	37	58,36	This work
C. jadinii CJ2	minimal media phytate	4,5*	50*	146	This work
T. delbrueckii CBS 1466	minimal media phytate	4,5	37	6,1	This work
K. marxianus Y1058	minimal media phytate	4,5	37	4,17	This work
K. marxianus Y1058	minimal media phytate	4,5	50*	7,11	This work
C. laurentii AL27	minimal media phytate	5	37	15.7	Pavlova et al., 2008
C. tropicalis NCIM 3321	MYGP	4,5*	70*	236	Puppala et al., 2018
C. tropicalis NCIM 3321	MYGP (different concentrations)	4,5*	70*	1014	Puppala et al., 2018
S. cerevisiae NCIM 3662	MYGP	4,5*	50*	45	Puppala et al., 2019
S. cerevisiae NCIM 3662	MYGP (different concentrations)	4,5*	50*	164	Puppala et al., 2019
W. anomalus	minimal media phytate	4	60	6	Vohra and Satyanarayana, 2001
W. anomalus	cane molasses (fed batch)	4	60	176	Vohra and Satyanarayana, 2004

MYGP: (Malt extract, Yeast extract, Glucose, Peptone). pH and temperature marked with * indicate optimum value.

2. Supplementary Figure

Figure S1. Kinetics of growth of yeasts MMPhy medium.

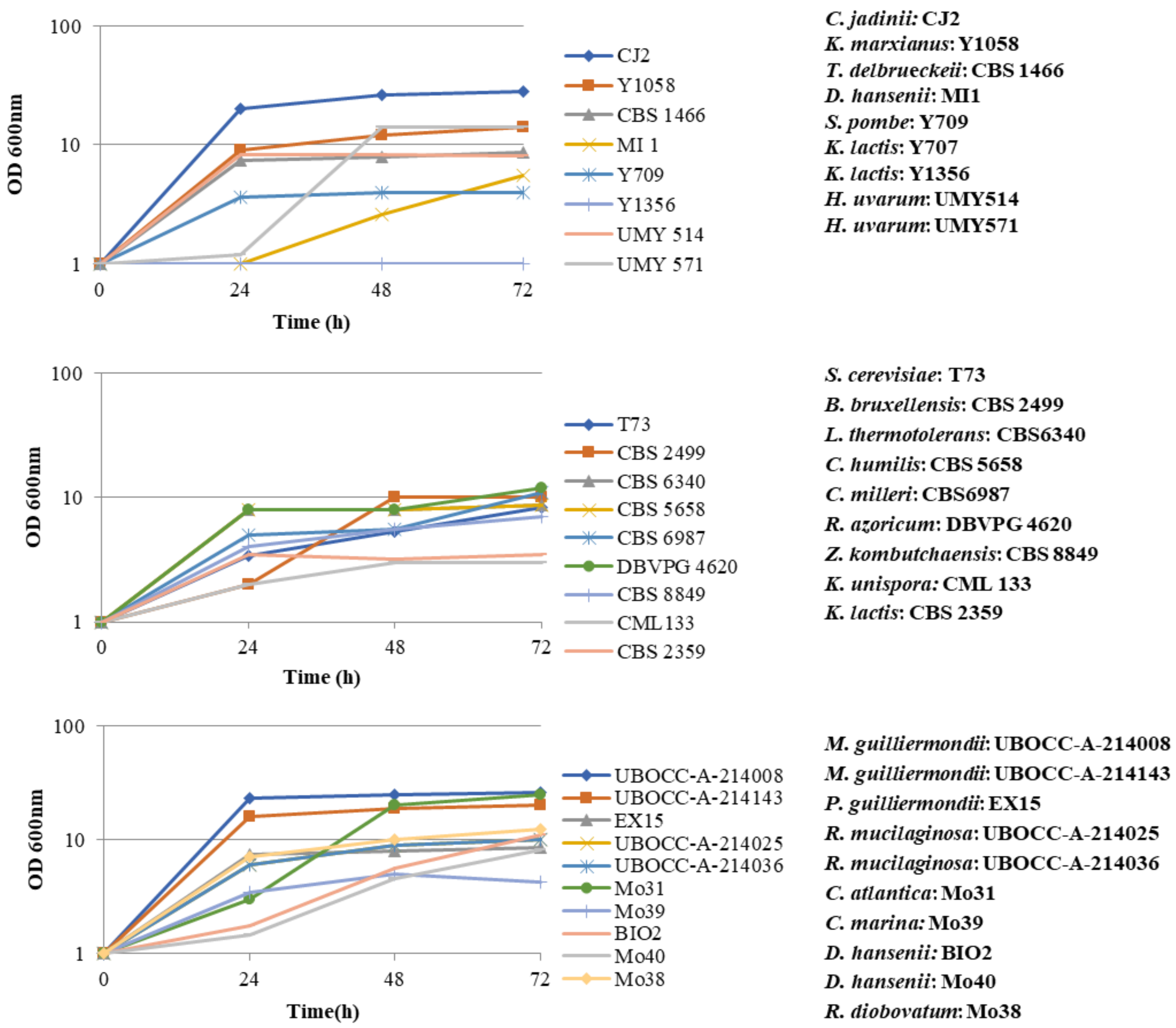

2 Supplementary references

Burgaud, G., Arzur, D., Durand, L., Cambon-Bonavita, M. A., and Barbier, G. (2010). Marine culturable yeasts in deep-sea hydrothermal vents: Species richness and association with fauna. FEMS Microbiol. Ecol. 73, 121–133. https://doi.org/10.1111/j.1574-6941.2010.00881.x

Burgaud, G., Hué, N. T. M., Arzur, D., Coton, M., Perrier-Cornet, J. M., Jebbar, M., et al. (2015). Effects of hydrostatic pressure on yeasts isolated from deep-sea hydrothermal vents. Res. Microbiol. 166, 700–709. https://doi.org/10.1016/j.resmic.2015.07.005

Capusoni, C., Rodighiero, V., Cucchetti, D., Galafassi, S., Bianchi, D., et al. (2017). Characterization of lipid accumulation and lipidome analysis in the oleaginous yeasts *Rhodosporidium azoricum* and *Trichosporon oleaginosus*. Bioresour. Technol. 238, 281–289. https://doi.org/10.1016/j.biortech.2017.03.188

Decimo, M., Quattrini, M., Ricci, G., Fortina, M. G., Brasca, M., Silvetti, T., et al. (2017). Evaluation of microbial consortia and chemical changes in spontaneous maize bran fermentation. AMB Express, 7:205. https://doi.org/10.1186/s13568-017-0506-y

Hagman, A., Säll, T., and Piškur, J. (2014). Analysis of the yeast short-term Crabtree effect and its origin. FEBS J., 281, 4805–4814. https://doi.org/10.1111/febs.13019

Kurtzman, C. P. (2003). Phylogenetic circumscription of *Saccharomyces*, *Kluyveromyces* and other members of the *Saccharomycetaceae*, and the proposal of the new genera *Lachancea*, *Nakaseomyces*, *Naumovia*, *Vanderwaltozyma* and *Zygotorulaspora*. FEMS Yeast Res. 4, 233–245. https://doi.org/10.1016/S1567-1356(03)00175-2

Kurtzman, C. P. (2011). “Discussion of teleomorphic and anamorphic ascomycetous yeasts and yeast-like taxa”. In: The Yeasts a Taxonomy study, 5th edition, ed. C. P. Kurtzman, J. W. Fell and T. Boekhout (Elsevier B.V) , 293–307. https://doi.org/10.1016/B978-0-444-52149-1.00013-6

Otero, J. M., Vongsangnak, W., Asadollahi, M. A., Olivares-Hernandes, R., Maury, J., Farinelli, L., et al. (2010). Whole genome sequencing of *Saccharomyces cerevisiae*: From genotype to phenotype for improved metabolic engineering applications. BMC Genom. 11:1. https://doi.org/10.1186/1471-2164-11-723

Pavlova, K., Gargova, S., Hristozova, T., and Tankova, Z. (2008). Phytase from antarctic yeast strain *Cryptococcus laurentii* AL27. Folia Microbiol. 53, 29–34. https://doi.org/10.1007/s12223-008-0004-3

Piškur, J., Ling, Z., Marcet-Houben, M., Ishchuk, O. P., Aerts, A., Labutti, K., et al. (2012). The genome of wine yeast *Dekkera bruxellensis* provides a tool to explore its food-related properties. Int. J. Food Microbiol. 157, 202–209. https://doi.org/10.1016/j.ijfoodmicro.2012.05.008

Puppala, K. R., Naik, T., Shaik, A., Dastager, S., Kumar V, R., Khire, J., et al. (2018). Evaluation of *Candida tropicalis* (NCIM 3321) extracellular phytase having plant growth promoting potential and process development. Biocatal. Agric. Biotechnol. 13, 225–235. https://doi.org/10.1016/j.bcab.2017.12.013

Puppala, K. R., Ravi Kumar, V., Khire, J., and Dharne, M. (2019). Dephytinizing and probiotic potentials of *Saccharomyces cerevisiae* (NCIM 3662) strain for amelioration of nutritional quality of functional foods. Probiotics Antimicrob. Proteins 11, 604–617. https://doi.org/10.1007/s12602-018-9394-y

Rédou, V., Navarri, M., Meslet-Cladière, L., Barbier, G., and Burgaud, G. (2015). Species richness and adaptation of marine fungi from deep-subseafloor sediments. Appl. Environ. Microbiol. 81, 3571–3583. https://doi.org/10.1128/AEM.04064-14

Vohra, A., and Satyanarayana, T. (2001). Phytase production by the yeast *Pichia anomala*. Biotechnol. Lett. 23, 551-554. https://doi.org/10.1023/A:1010314114053

Vohra, A., and Satyanarayana, T. (2004). A cost-effective cane molasses medium for enhanced cell bound phytase production by *Pichia anomala*. J. Appl. Microbiol. 97(3), 471–476. https://doi.org/10.1111/j.1365-2672.2004.02327.x

Wésolowski, M., Dumazert, P., and Fukuhara, H. (1982). Killer DNA plasmids of the yeast *Kluyveromyces lactis*. Curr. Genet. 5, 199–203. https://doi.org/10.1007/BF00391806

Yarrow, D. (1978). *Candida milleri* sp. nov. Int J Syst Bacteriol. 28, 608–610. https://doi.org/10.1099/00207713-28-4-608

Zhou, N., Ishchuk, O. P., Knecht, W., Compagno, C., and Piškur, J. (2019). Improvement of thermotolerance in *Lachancea thermotolerans* using a bacterial selection pressure. J. Ind. Microbiol. Biotechnol. 46, 133–145. https://doi.org/10.1007/s10295-018-2107-4
